# Prognosis of incidental pulmonary embolism vs. symptomatic pulmonary embolism in cancer patients: a single-center retrospective cohort study in China

**DOI:** 10.1186/s12959-023-00502-6

**Published:** 2023-06-06

**Authors:** Yanfei Wang, Zhongfen Liu, Qiuyu Li, Lina Xia, Yunyi Wang, Danfeng Jiang, Xiaoyan Chen, Yanqun Zheng, Wei Liu, Dan Wang, Dong Xue

**Affiliations:** 1 Peking University Cancer Hospital and Beijing Cancer Institute, Day Oncology Unit, Key Laboratory of Malignant Tumor Pathogenesis and Transformation Research, Ministry of Education, Ministry of Education, Beijing, China; 2 Peking University Cancer Hospital and Beijing Cancer Institute, Department of Supportive Care, Key Laboratory of Malignant Tumor Pathogenesis and Transformation Research, Ministry of Education, Beijing, China; 3 Peking University Third Hospital, Department of Respiratory and Critical Care Medicine, Beijing, China; 4 Peking University Cancer Hospital and Beijing Cancer Institute, Medical Department, Key Laboratory of Malignant Tumor Pathogenesis and Transformation Research, Ministry of Education, Beijing, China; 5 Peking University Cancer Hospital and Beijing Cancer Institute, Department of Integrated Traditional Chinese and Western Medicine, Key Laboratory of Malignant Tumor Pathogenesis and Transformation Research, Ministry of Education, 100142 Beijing, China

**Keywords:** Cancer, Pulmonary embolism, Thrombosis, Prognosis, Survival

## Abstract

**Background:**

The incidence of incidental pulmonary embolism (IPE) has greatly increased, but its clinical characteristics and outcomes are still controversial. This study aimed to compare the clinical characteristics and outcomes between cancer patients with IPE and patients with symptomatic pulmonary embolism (SPE).

**Patients/Methods:**

Clinical data of 180 consecutive patients with cancer complicated with pulmonary embolism admitted to Beijing Cancer Hospital from July 2011 to December 2019 were retrospectively collected and analysed. General characteristics, diagnosis time of pulmonary embolism (PE), location of PE, concurrent deep venous thrombosis, anticoagulant treatment, impact of PE on anti-tumor treatment, recurrent venous thromboembolism, rate of bleeding after anticoagulation therapy, survival and risk factors of IPE were compared with SPE.

**Results:**

Of 180 patients, 88 (49%) had IPEs and 92 (51%) had SPEs. Patients with IPE and SPE did not differ in age, sex, tumor type, or tumor stage. Median diagnosis times of IPE and SPE after cancer were 108 (45, 432) days and 90 (7, 383) days, respectively. Compared to SPE, IPE tended to be central (44% versus 26%; P < 0.001), isolated (31.8% versus 0.0%; P < 0.001), and unilateral (67.1% versus 12.8%; P < 0.00). The rate of bleeding after anticoagulation therapy did not differ between IPE and SPE. Patients with IPE had a better prognosis than patients with SPE in terms of 30-, and 90-day mortality, as well as overall survival after diagnosis of PE (median: 314.5 vs. 192.0 days, log-rank P = 0.004) and cancer (median: 630.0 vs. 450.5 days, log-rank P = 0.018). SPE (compared to IPE) was an independent risk factor for poor survival after diagnosis of PE in multivariate analysis (hazard ratio [HR] = 1.564, 95% confidence interval [CI]: 1.008–2.425, p = 0.046).

**Conclusions:**

IPE accounts for nearly one half of PE cases among Chinese cancer patients. With active anticoagulation treatment, IPE is expected to achieve better survival rates than SPE.

## Introduction

Incidental pulmonary embolism (IPE) is defined as clinically unsuspected filling defects of the pulmonary arteries found on imaging performed for purposes other than suspicion of pulmonary embolism (PE) [1]. With the growing use and improvement of scanners and imaging qualities of computed tomography (CT) in recent years, the frequency of IPE has increased [2]. Patients with cancer are at a high risk of venous thromboembolism (VTE), including PE [3]. In addition, patients with cancer frequently undergo chest CT examinations for cancer diagnosis, staging, and treatment efficacy evaluation; thus, IPE is found even more prevalently in patients with cancer [4]. IPE has been found in 3.3–5.0% of patients with cancer and detected in 1–2% of all thoracic CT examinations [1, 5–8]. Many IPEs are symptomatic, although the symptoms can be missed or misattributed [9]. Therefore, the International Society on Thrombosis and Hemostasis recommended using the term “incidental” instead of “asymptomatic” [10].

Despite the common occurrence of IPE, the clinical characteristics and outcomes of this patient population are still controversial, studies on IPE revolving around treatment and outcome are limited, and the optimal management in patients remains unclear [9]. Therefore, we sought to identify the clinical characteristics and describe the treatment and clinical outcomes of patients with cancer complicated by IPE and symptomatic pulmonary embolism (SPE).

## Patients and methods

### Study design and population

This single-center retrospective cohort study was conducted at Peking University Cancer Hospital, a tertiary teaching hospital that treats patients with cancer from all over China.

We identified all patients with cancer who visited the Peking University Cancer Hospital and whose electronic medical records included International Classification of Diseases codes for PE. In addition, we cross-referenced the radiology database to identify those who had been suggested as having PE based on CT findings to avoid omission of relevant patients. Patients were included if they met all the following inclusion criteria: (1) diagnosis of a malignant tumor by histopathological examination; (2) age ≥ 18 years; (3) diagnosis of PE was made in the electronic medical record or CT report; and (4) admission to Peking University Cancer Hospital from July 2011 to December 2019. Patients were excluded if (1) surgery was performed within 30 days prior to the diagnosis of PE; (2) a diagnosis of PE was made based on clinical experience, without imaging confirmation by contrast-enhanced chest CT or CT pulmonary angiography (CTPA); and (3) patients had incomplete medical records.

**Fig. 1:**
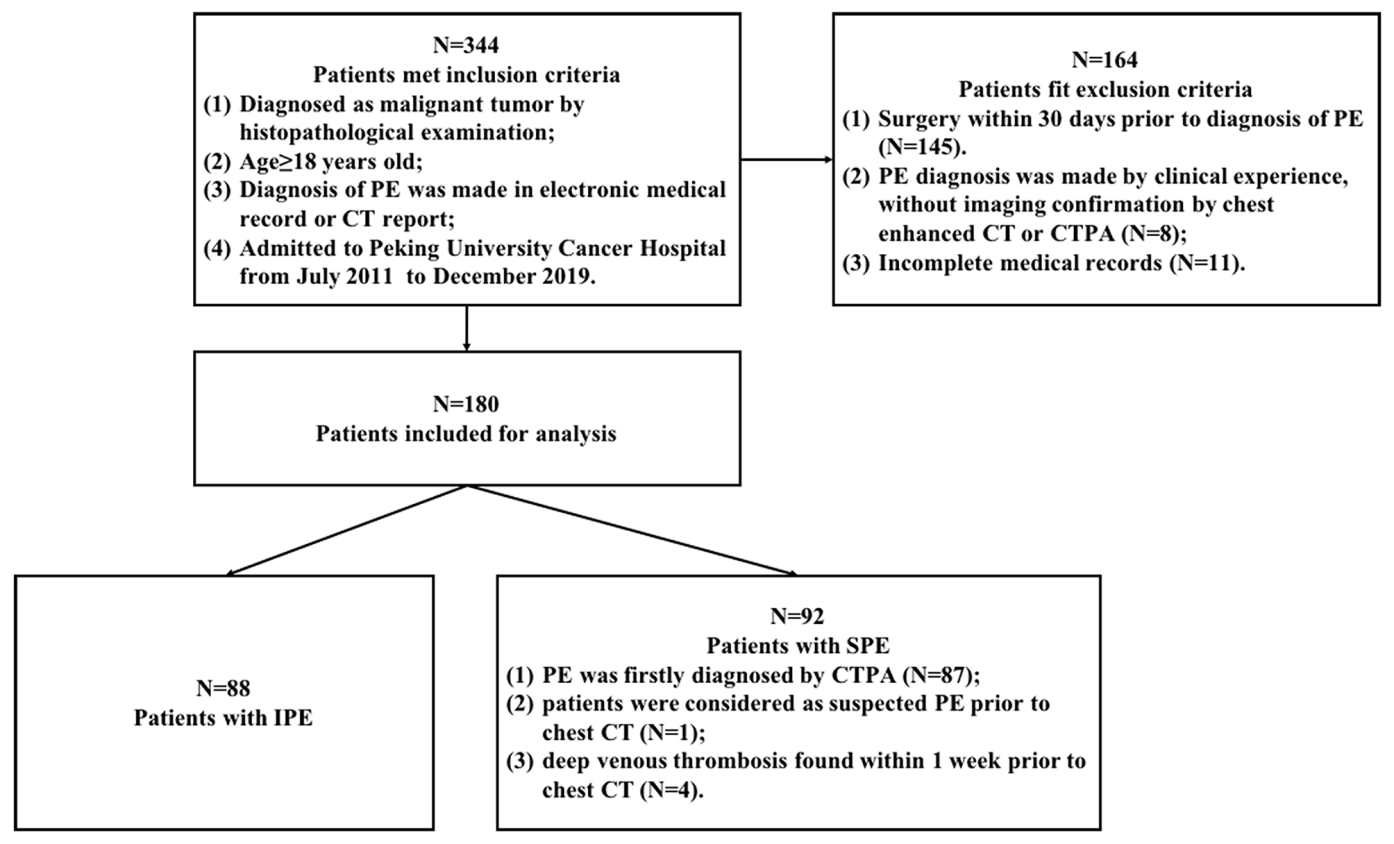
Study flowchart PE: pulmonary embolism; CT: computed tomography; CTPA: computed tomography pulmonary angiography; IPE: incidental pulmonary embolism; SPE: symptomatic pulmonary embolism

From the database, 344 records were retrieved, of which 180 were included in this study (Fig. 1).

### Identification of IPE and SPE

Patients with PE were identified as having SPE if they met any of the following conditions: (1) PE was first diagnosed by CTPA; (2) patients were considered to have suspected PE prior to chest CT findings recorded in the electronic medical records; and (3) deep venous thrombosis (DVT) was found within 1 week before chest CT. Patients with PE who met none of the aforementioned criteria were identified as having IPE, regardless of the presence of symptoms. Cases with missing data were excluded for data analysis.

Finally, 88 patients were diagnosed with IPE, whereas 92 patients were diagnosed with SPE (Fig. 1).

### Data collection and definitions

Clinical data of each patient, including sex, age, location of the primary tumor, tumor stage, anti-tumor treatment, specific manifestations of PE, PE location, concurrent DVT, anticoagulant treatment, impact of PE on anti-tumor treatment, recurrent VTE, bleeding events, and survival, were collected by reviewing patients’ electronic medical records. Clinical data were double recorded and cross-examined by two physicians to ensure the precision and completion.

The time from cancer diagnosis was calculated from the time of the confirmed pathological diagnosis to the time of the radiological diagnosis of PE.

A patient was considered to be in the active stage of cancer if any of the following applied [11]: (1) the patient did not receive possible curative treatment; (2) there was evidence that the treatment did not cure the disease (e.g., recurrent or progressive disease); or (3) the treatment was ongoing.

Active anti-cancer treatment referred to anti-cancer treatment received within 1 month before the diagnosis of PE.

Bleeding events included skin bleeding (ecchymosis), gastrointestinal bleeding (hematemesis, hematochezia, and positive fecal occult blood), hemoptysis, and intracerebral hemorrhage. Massive bleeding was defined as meeting any of the following [12]: (1) fatal bleeding; (2) symptomatic bleeding in key areas or organs, such as intracranial, intraspinal, intraocular, retroperitoneal, and intra-articular or pericardial, or osteo-fascial compartment syndrome caused by bleeding; and (3) bleeding that results in a decrease in the hemoglobin level of ≥ 20 g/L or infusion of ≥ 2 units of whole blood or red blood cells.

Overall survival was defined as the time from radiological diagnosis of PE to death, with censoring for survival or lost to follow-up.

### Statistical analysis

Normally distributed continuous variables are presented as means and standard deviations; otherwise, they are presented as medians and interquartile ranges (IQR). Categorical variables are presented as counts and percentages. The one-sample Kolmogorov–Smirnov test was used to determine whether the data had a normal distribution. Inter-group comparisons between continuous variables were made using the Student t-test or Wilcoxon rank-sum test, whereas comparisons of categorical variables between the IPE and SPE groups were made using the χ^2^ test. Kaplan–Meier survival analysis followed by the log-rank test was used to estimate the difference in overall survival between the groups. Lost to follow-up was addressed as a censor. Potential risk factors for the outcomes were evaluated using the Cox proportional hazards model. Univariate and multivariate Cox hazard analyses were performed to analyze overall survival. Parameters with a P value < 0.1 were included in multivariate analysis of survival. When 2 parameters were correlated with each other, only 1 parameter was included.

All analyses were performed using the Software Package for Social Sciences (SPSS, version 21.0; IBM Corp., Armonk, NY). Statistical significance was set at two-tailed P-value < 0.05.

## Results

### Patient characteristics

From July 2011 to December 2019, 180 patients with PE were included in this study, of which 88 (49%) had IPE and 92 (51%) had SPE. Table 1 shows the general characteristics of patients in each group. The ages of patients with IPE and SPE were similar (median age [IQR]: 61 [54–66] years versus [vs.] 60 [53–68] years; P = 0.934). In addition, patients with IPE and SPE did not differ in sex, the presence of 2 primary tumors, tumor type, or tumor stage. All patients were with active cancer.

**Table 1:** of General characteristics patients with cancer complicated with IPE and SPE.

Characteristics	IPE, N = 88	SPE, N = 92	P-value
Age, median (IQR), y	61.0 (54–66)	60 (53–68)	0.934
Sex			0.095
Female	35 (39.8)	44 (47.8)	
Male	53 (60.2)	48 (52.2)	
Location of the primary tumor			0.298
Lung	33 (37.5)	47 (51.1)	
Digestive	17 (19.3)	16 (17.4)	
Breast	7 (7.9)	10 (10.9)	
Lymphoma	12 (13.6)	6 (6.5)	
Melanoma	8 (9.1)	4 (4.3)	
Urogenital	8 (9.1)	8 (8.7)	
Others	3 (3.4)	1 (1.1)	
Cancer stage			0.511
I	3 (3.4)	3 (3.3)	
II	4 (4.5)	1 (1.1)	
III	13 (14.8)	17 (18.5)	
IV	68 (77.3)	71 (77.2)	
ECOG PS			< 0.001^*^
0	29 (33.0)	8 (8.7)	
1	48 (54.5)	43 (46.7)	
2	9 (10.2)	26 (28.3)	
3	2 (2.3)	10 (10.9)	
4	0 (0.0)	5 (5.4)	
Cancer treatment before PE			
Cytotoxic chemotherapy	60 (68.2)	53 (57.6)	0.142
Targeted chemotherapy	29 (32.9)	26 (28.3)	0.494
Immunotherapy	4 (4.5)	4 (4.3)	0.436
Hormonal therapy	4 (4.5)	8 (8.7)	0.264
Multiple treatment regimens	31 (35.2)	31 (33.7)	0.829
Radiotherapy	14 (15.9)	18 (19.6)	0.622
Active cancer treatment before PE			
Cytotoxic chemotherapy	48 (54.5)	42 (45.6)	0.233
Targeted chemotherapy	20 (22.7)	24 (26.1)	0.600
Immunotherapy	2 (2.3)	4 (4.3)	0.683
Hormonal therapy	1 (1.1)	3 (3.3)	0.621
Multiple treatment regimens	15 (17.0)	12 (13.0)	0.452
Radiotherapy	1 (1.1)	0 (0.0)	0.489

Unless otherwise noted, data are presented as n (%)

IPE, incidental pulmonary embolism; SPE, symptomatic pulmonary embolism; IQR, interquartile range; PE, pulmonary embolism; ECOG PS, Eastern Cooperative Oncology Group performance status; * means P < 0.05

### Time of PE diagnosis after cancer

The median times from cancer diagnosis to PE diagnosis were 108 (IQR: 45–432) days in patients with IPE and 90 (IQR: 7–383) days in patients with SPE. More SPEs developed before cancer diagnosis than IPEs (15% vs. 2%; P = 0.00), as shown in Table 2. The median times from the initiation of systematic anti-tumor therapy to PE diagnosis were 81 (IQR: 9–421) days in patients with IPE and 90 (IQR: 7–383) days in patients with SPE.

### Symptoms of PE

Patients with IPE presented with PE-related symptoms much less frequently than those with SPE did (Table 2).

### Location of PE

The rates of concurrent DVT were similar between the IPE and SPE groups (52.3% and 47.7%, respectively; P = 0.551). However, the location of PE differed between the groups. Compared with SPE, IPE tended to be central, isolated, and unilateral (Table 2).

**Table 2:** Clinical characteristics of IPE and SPE.

Characteristics	IPE	SPE	P-value
*Time of diagnosis*			
	N = 88	N = 92	
Before cancer diagnosis	2 (2.3)	14 (15.2)	0.00^*^
	N = 86	N = 78	
Within 6 months after cancer diagnosis	54 (62.8)	43 (55.1)	0.32
Within 2 years after cancer diagnosis	73 (84.9)	64 (82.0)	0.63
*Symptom*	N = 88	N = 92	
Hemoptysis	0 (0.0)	6 (6.5)	0.029^*^
Dyspnea	4 (4.5)	68 (73.9)	<0.001^*^
Cough	2 (2.3)	26 (28.3)	< 0.001^*^
Fatigue	5 (5.7)	30 (32.6)	< 0.001^*^
Chest pain	1 (1.1)	21 (22.8)	< 0.001^*^
*Location*	N = 88	N = 92	
Main or lobar	55 (62.5)	10 (10.9)	< 0.001^*^
Main, lobar, or segmental	84 (95.4)	40 (43.5)	< 0.001^*^
Multiple	60 (68.2)	92 (100.0)	< 0.001^*^
Bilateral	29 (32.9)	71 (77.2)	< 0.001^*^

Data are presented as n (%)

IPE, incidental pulmonary embolism; SPE, symptomatic pulmonary embolism; PE, pulmonary embolism; * means P < 0.05

### Patients with IPE and SPE received similar anticoagulation treatment

The proportions of patients who received anticoagulation therapy were similar between the groups, although more patients with SPE received low molecular weight heparin (LMWH) as an anticoagulant drug in the initial phase and the entire anticoagulant course than those with IPE (Table 3).

**Table 3:** Treatment of PE and Effect of PE on anti-cancer treatment

	IPE, N = 88	SPE, N = 92	P-value
	N = 83	N = 91	
Anticoagulation	81 (97.6)	90 (98.9)	0.465
	N = 78	N = 90	
*Initial anticoagulant treatment*			
LMWH	61 (78.2)	80 (88.9)	0.004^*^
Unfractionated heparin	0 (0.0)	2 (2.2)	0.260
Warfarin	9 (11.5)	6 (6.7)	0.369
Rivaroxaban	8 (10.3)	2 (2.2)	0.043*
*Whole anticoagulation course*			
LMWH	65 (83.3)	79 (87.8)	0.044^*^
Unfractionated heparin	0 (0.0)	3 (3.3)	0.131
Warfarin	12 (15.4)	14 (15.6)	0.933
Rivaroxaban	13 (16.7)	9 (10.0)	0.307
	N = 88	N = 92	
IVC filter placement	9 (10.2)	6 (6.5)	0.356
Thrombolytic therapy	2 (2.3)	3 (3.3)	0.521
	N = 66	N = 73	
Standard dosage of LMWH	46 (69.7)	54 (74.0)	0.706

Data are presented as n (%)

IPE, incidental pulmonary embolism; SPE, symptomatic pulmonary embolism; PE, pulmonary embolism; LMWH, low molecular weight heparin; IVC, inferior vena cava; * means P < 0.05

### Effect of PE on anti-tumor therapy

In both groups, > 40% of the patients who received anti-tumor therapy regimens were affected by PE (Table 4). However, patients with IPE had higher rates of anti-tumor therapy delay, whereas patients with SPE had higher rates of anti-tumor therapy termination.

**Table 4:** Effect of PE on anti-cancer treatment

	IPE, N = 88	SPE, N = 92	P-value
Affected	38 (43.2)	42 (45.6)	0.640
Delayed	16 (18.2)	4 (4.7)	0.003*
Dosage decreased	1 (1.1)	1 (1.1)	0.740
Stopped	13 (14.8)	25 (27.2)	0.042*
Changed	9 (10.2)	12 (13.0)	0.556

Data are presented as n (%)

IPE, incidental pulmonary embolism; SPE, symptomatic pulmonary embolism; PE, pulmonary embolism; * means P < 0.05

### Prognosis of PE

Patients were followed up by routine outpatient services and telephone. Within a median follow-up time of 237.5 (IQR: 82.25–620.25) days after diagnosis of PE, bleeding events and recurrent VTE occurred in 18 (10.0%) and 15 (8.3%) patients respectively, and 93 (51.7%) patients died.

Follow-up chest CT/CTPA and/or vascular ultrasonography was performed in 69 (78.4%) patients with IPE and 46 (50.0%) with SPE. The rates of recurrent VTE were 7.2% (5/69) and 21.7% (10/46) in the patients with IPE and SPE, respectively (Table 5).

Significant differences were observed between patients with IPE and SPE in terms of 30-, and 90-day mortality, with better short-term survival in patients with IPE than in those with SPE (Table 5).

**Table 5:** Outcomes of cancer patients with IPE and SPE.

	IPE	SPE	P-value
All patients	N = 88	N = 92	
Follow-up time, median, IQR, d	314.5 (141.25–620.25)	192 (54.25–544.5)	0.033^*^
Bleeding events	8 (9.1)	10 (10.9)	0.691
Recurrent VTE	5 (5.7)	10 (10.9)	0.208
Underwent follow-up radiology	69 (78.4)	46 (50.0)	0.001^*^
Mortality	36 (40.9)	57 (62.0)	0.005^*^
Mortality in 30 days	0 (0.0)	9 (9.8)	0.003^*^
Mortality in 90 days	3 (3.4)	18 (19.6)	0.001^*^
Patients who underwent follow-up radiology	N = 69	N = 46	
Recurrent VTE	5 (7.2)	10 (21.7)	0.024^*^

Unless otherwise noted, data are presented as n (%)

IPE, incidental pulmonary embolism; SPE, symptomatic pulmonary embolism; PE, pulmonary embolism; IQR, interquartile range; VTE, venous thromboembolism; * means P < 0.05

**Fig. 2:**
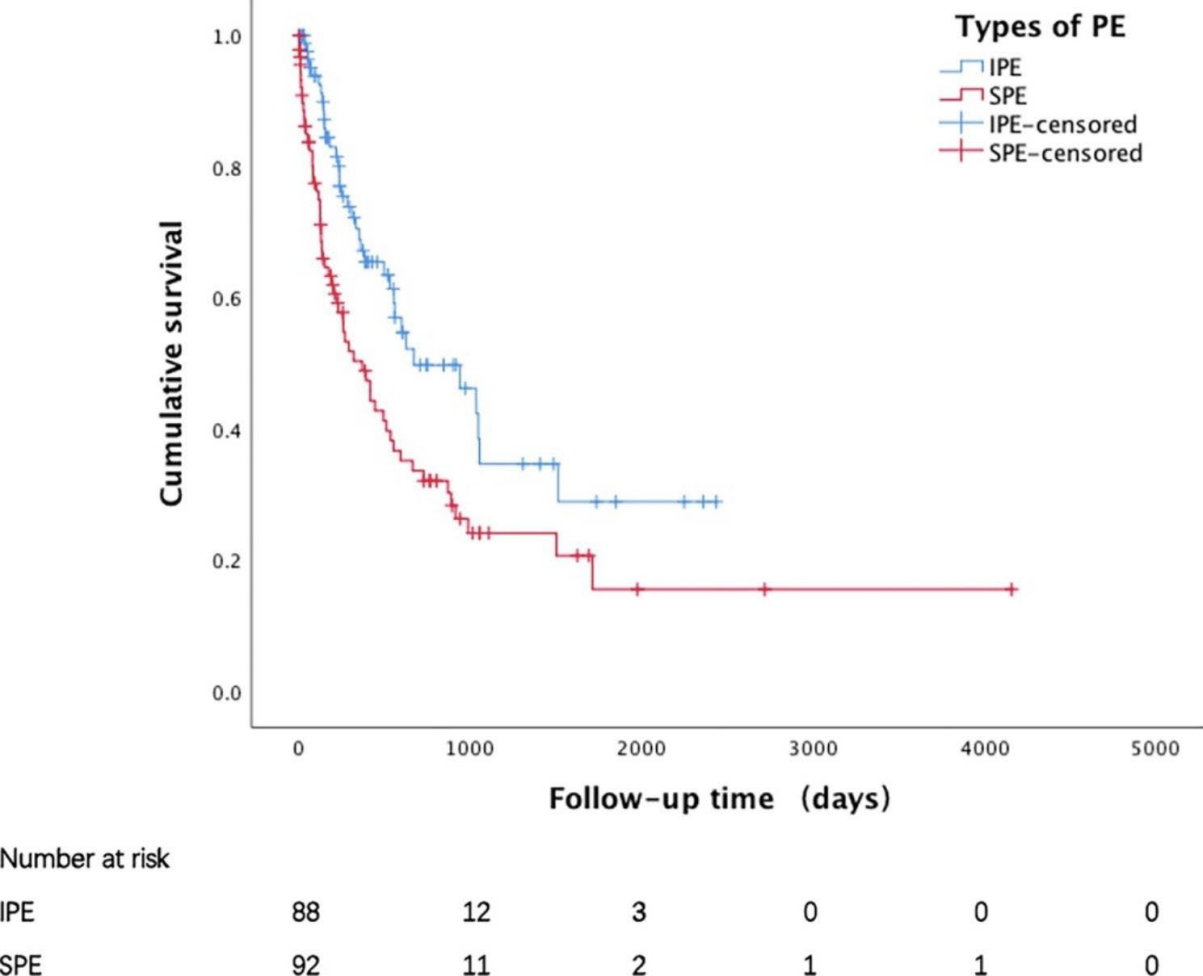
Overall survival of patients with IPE and SPE after the diagnosis of pulmonary embolism IPE, incidental pulmonary embolism; SPE, symptomatic pulmonary embolism

**Fig. 3:**
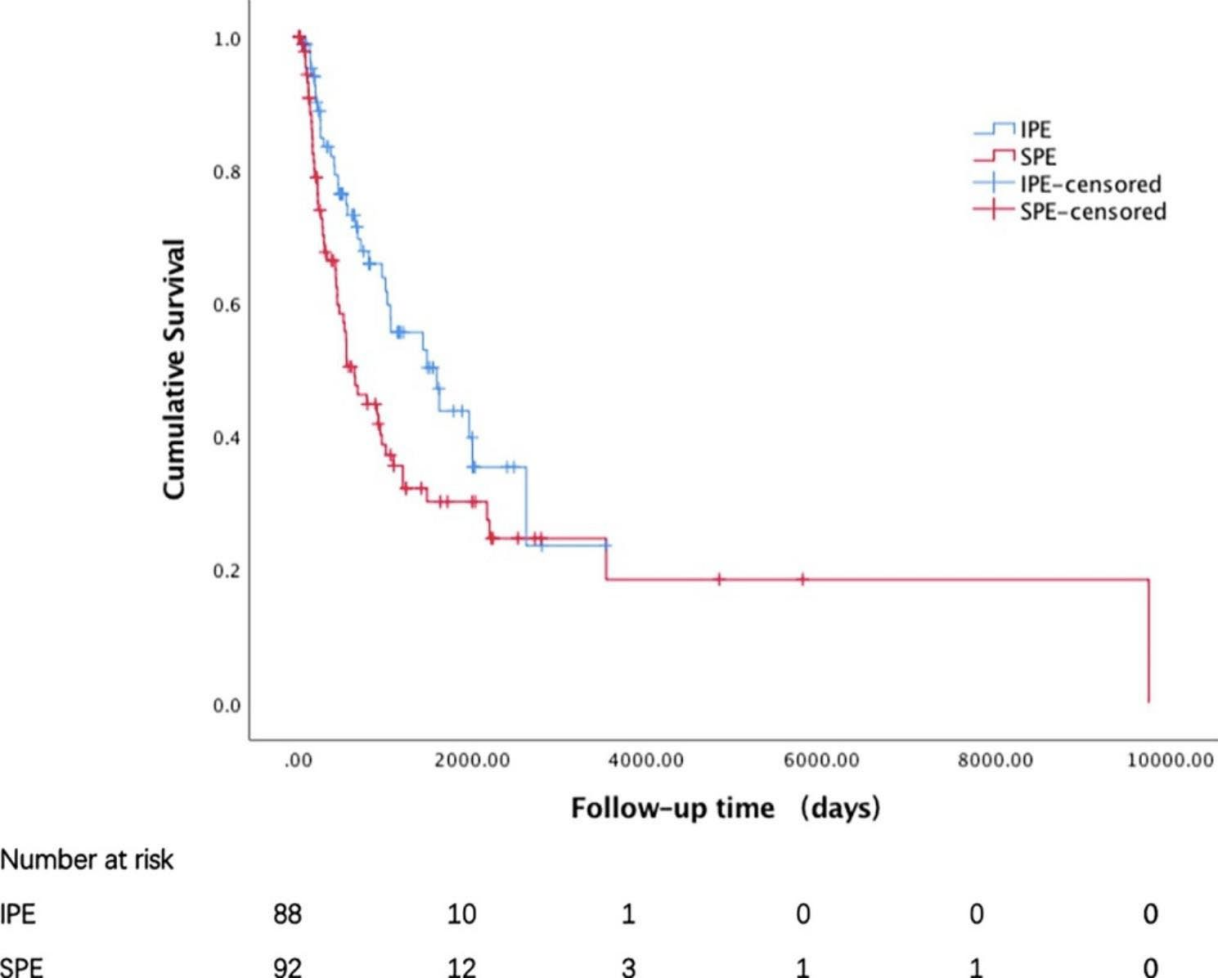
Overall survival of patients with IPE and SPE after the diagnosis of cancer IPE, incidental pulmonary embolism; SPE, symptomatic pulmonary embolism

Kaplan–Meier analysis showed that IPE had significantly better prognosis than SPE in terms of overall survival after diagnosis of PE (median: 314.5 vs. 192.0 days, P = 0.005) (Fig. 2), as well as diagnosis of cancer (median: 630.0 vs. 450.5 days, P = 0.018) (Fig. 3).

Univariate Cox hazard survival analysis showed that the risk factors for shorter survival after diagnosis of PE in patients with cancer included age ≥ 70 years, stage IV disease, symptomatic PE, Eastern Cooperative Oncology Group performance status (ECOG PS) > 1, and thrombolytic therapy, whereas being overweight was shown to be a protective factor. In further multivariate Cox hazard analysis of the risk factors, age ≥ 70 years, stage IV disease, symptomatic PE, ECOG PS > 1, and thrombolytic therapy were shown to be independent risk factors for overall survival after adjustment using a stepwise model (Table 6).

**Table 6:** Univariate and multivariate Cox hazard analyses of the risk factors for survival after diagnosis of PE

	Univariate analysis		Multivariate analysis	
	HR (95% CI)	P-value	HR (95% CI)	P-value
Age ≥ 70 y	1.818 (1.116–2.962)	0.016^*^	2.487 (1.487–4.161)	0.001^*^
Female sex	1.038 (0.690–1.562)	0.857		
Stage IV disease	2.028 (1.165–3.531)	0.012^*^	2.024 (1.131–3.623)	0.018^*^
SPE	1.823 (1.200–2.768)	0.005^*^	1.564 (1.008–2.425)	0.046^*^
Central or lobar location	0.808 (0.520–1.254)	0.342		
Central, lobar, or segmental location	0.919 (0.599–1.411)	0.700		
Multiple	1.578 (0.908–2.745)	0.106		
Bilateral	1.203 (0.793–1.823)	0.385		
Combined with DVT	1.184 (0.787–1.781)	0.418		
ECOG PS > 1	2.784 (1.821–4.258)	< 0.001^*^	2.276 (1.429–3.626)	0.001^*^
Low BMI	1.329 (0.539–3.278)	0.537		
Overweight	0.663 (0.441–0.997)	0.048^*^	0.896 (0.590–1.359)	0.604
Obesity	0.687 (0.374–1.262)	0.226		
Anticoagulation therapy	0.834 (0.515–1.353)	0.463		
Thrombolytic therapy	3.762 (1.342–10.545)	0.012^*^	5.208 (1.801–15.063)	0.002^*^
Filter implantation	0.654 (0.286–1.497)	0.315		
Bleeding event	0.909 (0.468–1.765)	0.778		

PE, pulmonary embolism; HR, hazard ratio; CI, confidence interval; SPE, symptomatic pulmonary embolism; DVT, deep vein thrombosis; ECOG PS, Eastern Cooperative Oncology Group performance status; BMI, body mass index; * means P < 0.05

For patients with IPE, a separate analysis revealed that overweight was identified as the only independent protective factor for overall survival in multivariate analysis (Table 7).

**Table 7:** Univariate and multivariate Cox hazard analysis of the risk factors for survival after diagnosis of IPE.

	Univariate analysis		Multivariate analysis	
	HR (95% CI)	P-value	HR (95% CI)	P-value
Age ≥ 70 y	0.864 (0.335–2.229)	0.763		
Female sex	1.019 (0.523–1.984)	0.857		
Stage IV disease	3.469 (1.217–9.890)	0.020^*^	2.887 (0.998–8.347)	0.050
Central or lobar location	1.009 (0.519–1.961)	0.979		
Central, lobar, or segmental location	1.808 (0.246–13.316)	0.561		
Multiple	1.170 (0.574–2.384)	0.106		
Bilateral	1.131 (0.572–2.236)	0.723		
Combined with DVT	1.182 (0.611–2.287)	0.620		
ECOG PS > 1	2.901 (1.310–6.426)	0.009^*^	1.779 (0.766–4.134)	0.181
Low BMI	0.048 (0.000–6908.772)	0.617		
Overweight	0.402 (0.205–0.786)	0.008^*^	0.490 (0.245–0.983)	0.045*
Obesity	0.734 (0.284–1.897)	0.523		
Anticoagulation therapy	1.322 (0.633–2.763)	0.457		
Thrombolytic therapy	4.845 (0.611–38.452)	0.135		
Filter implantation	0.493 (0.118–2.057)	0.332		
Bleeding event	0.585 (0.178–1.916)	0.375		

PE, pulmonary embolism; HR, hazard ratio; CI, confidence interval; DVT, deep vein thrombosis; ECOG PS, Eastern Cooperative Oncology Group performance status; BMI, body mass index; * means P < 0.05

For patients with SPE, on the other hand, age ≥ 70 years, ECOG PS > 1, and thrombolytic therapy were identified as independent risk factors for poor overall survival in multivariate analysis (Table 8).

**Table 8:** Univariate and multivariate Cox hazard analysis of the risk factors for survival after diagnosis of SPE

	Univariate analysis		Multivariate analysis	
	HR (95% CI)	P-value	HR (95% CI)	P-value
Age ≥ 70 y	2.624 (1.455–4.768)	0.001^*^	3.141 (1.691–5.836)	< 0.001^*^
Female sex	0.897 (0.532–1.511)	0.683		
Stage IV disease	1.552 (0.801–3.009)	0.193		
Central or lobar location	1.437 (0.678–3.045)	0.345		
Central, lobar, or segmental location	1.482 (0.878–2.503)	0.141		
Bilateral	0.682 (0.372–1.251)	0.216		
Combined with DVT	1.191 (0.706–2.010)	0.513		
ECOG PS > 1	2.285 (1.339–3.899)	0.002^*^	2.557 (1.474–4.437)	0.001*
Low BMI	1.272 (0.505–3.205)	0.610		
Overweight	0.967 (0.574–1.628)	0.899		
Obesity	0.678 (0.304–1.509)	0.341		
Anticoagulation therapy	0.671 (0.339–1.327)	0.251		
Thrombolytic therapy	3.001 (0.913–9.868)	0.070	4.940 (1.446–16.878)	0.011^*^
Filter implantation	0.924 (0.333–2.564)	0.880		
Bleeding event	1.206 (0.536–2.715)	0.651		

PE, pulmonary embolism; HR, hazard ratio; CI, confidence interval; DVT, deep vein thrombosis; ECOG PS, Eastern Cooperative Oncology Group performance status; BMI, body mass index; * means P < 0.05

Risk factors influencing survival after diagnosis of cancer were also evaluated. Univariate Cox hazard survival analysis showed that the risk factors for shorter survival after diagnosis of cancer in cancer patients with PE included age ≥ 70 years, stage IV disease, SPE, and ECOG PS > 1, whereas being overweight was shown to be a protective factor. In further multivariate Cox hazard analysis of the risk factors, age ≥ 70 years and ECOG PS > 1 were shown to be independent risk factors, while overweight was shown to be an independent protective factor for overall survival after adjustment using a stepwise model (Table 9).

**Table 9:** Univariate and multivariate Cox hazard analyses of the risk factors for survival after diagnosis of cancer

	Univariate analysis		Multivariate analysis	
	HR (95% CI)	P-value	HR (95% CI)	P-value
Age ≥ 70 y	1.989 (1.217–3.251)	0.006^*^	1.909 (1.152–3.162)	0.012^*^
Female sex	0.857 (0.567–1.296)	0.466		
Stage IV disease	1.765 (1.015–3.072)	0.044^*^	1.560 (0.879–2.768)	0.128
SPE	1.823 (1.200–2.768)	0.005^*^	1.341 (0.852–2.110)	0.205
Central or lobar location	0.775 (0.498–1.206)	0.258		
Central, lobar, or segmental location	0.902 (0.584–1.393)	0.643		
Multiple	1.431 (0.760–2.691)	0.267		
Bilateral	1.142 (0.751–1.738)	0.535		
Combined with DVT	1.029 (0.684–1.549)	0.891		
ECOG PS > 1	2.193 (1.436–3.350)	< 0.001^*^	1.702 (1.071–2.705)	0.025*
Low BMI	1.923 (0.779–4.750)	0.156		
Overweight	0.557 (0.369–0.841)	0.005^*^	0.647 (0.425–0.985)	0.042*
Obesity	0.709 (0.386–1.302)	0.267		
Anticoagulation therapy	20.698 (0.017–24612.726)	0.402		
Thrombolytic therapy	1.303 (0.470–3.616)	0.611		
Filter implantation	0.733 (0.320–1.682)	0.464		
Bleeding event	0.776 (0.399–1.510)	0.456		

PE, pulmonary embolism; HR, hazard ratio; CI, confidence interval; SPE, symptomatic pulmonary embolism; DVT, deep vein thrombosis; ECOG PS, Eastern Cooperative Oncology Group performance status; BMI, body mass index; * means P < 0.05

## Discussion

Recently, the diagnosis of VTE in patients with cancer has been increasing [13], while mortality has decreased [14]. Owing to the progress in radiological technology, regular CT scanning in patients with cancer facilitates the incidental diagnosis of PE. Our study showed that IPE occurred in nearly half of all PE patients with cancer, which is consistent with findings of previous studies [15, 16].

VTE affects the progress of anti-tumor treatment and increases mortality [17–19]. In this study, anti-tumor therapies in patients with IPE and SPE were affected in similar proportion. However, they were not affected in the same manner or to the same extent. More patients with IPE had delayed anti-cancer treatment than those with SPE, and many more patients with SPE than those with IPE had their treatment stopped. An explanation why patients with IPE were more likely to have a delay in anti-cancer treatment compared to SPE was because a majority of IPE patients get diagnosed by scheduled re-staging CT scans a couple of days within the expected subsequent therapy cycle, and therefore delayed in treatment in order to deal with the new thrombotic event, while SPE patients might get diagnosed at any time during the treatment cycle with presence of symptoms. Besides, the differences may be partly explained by the fact that more patients with SPE died shortly after the diagnosis of PE; thus, the delay in anti-tumor treatment turned out to be termination of anti-cancer therapy. Additionally, since the proportion of anti-tumor therapy stopped was bigger than that of short-term death, the difference in anti-tumor treatment strategies reflected the different attitudes of health care providers towards the two groups.

PE is a potentially fatal condition. Moreover, mortality in PE patients with cancer is higher than that in those without cancer [20, 21]. According to previous studies, there are no major differences in the risks of mortality between incidental and symptomatic venous thrombosis and between IPE and SPE [15, 22].

A recent meta-analysis [23] included three randomized controlled trials [24–26] to evaluate outcomes between cancer patients with incidental VTE and symptomatic VTE; it showed that incidental VTE presented with a significantly lower rate of recurrent VTE (relative risk [RR], 0.62; 95% CI: 0.44–0.87, P = 0.007) and a tendency to be higher in rate of major bleeding events but there was no statistical difference (RR, 1.47; 95% CI: 0.99–2.20, P = 0.06) than symptomatic VTE at 6 months [23]. There was no difference in overall mortality between the incidental and symptomatic VTE groups. It should be noted that the aforementioned findings were observed in patients with VTE, in whom PE accounted for 61.3%. As PE is a more serious disease than DVT, the former conclusion may not directly apply to PE.

Studies focusing on the differences between IPE and SPE are scarce. The EPIPHANY study was a multicenter observational study conducted in Spain focusing on short term survival of patients with cancer-associated PE. A subgroup prospective cohort of 497 patients indicated that SPE was shown as associated with the overall 30-day mortality on multivariate analysis, while patients with asymptomatic IPE had a significantly lower rate of 30- and 90-day mortality compared to those with symptomatic IPE and SPE (3% vs. 20% vs. 21%, P < 0.001; 12% vs. 43% vs. 33%, P < 0.001)[27]. There were no differences in 30- and 90-day VTE recurrence and major bleeding rates between IPE and SPE patients[27]. Our study confirmed the survival results of this prospective study. A Netherlands study suggested that patients with cancer diagnosed with IPE and those with SPE had no difference in the rates of recurrent VTE (13.3% vs. 16.9%, P = 0.77), major bleeding events (12.5% vs. 8.6%, P = 0.5), or 12-month mortality (52.9 vs. 53.3%, P = 0.7) [22]. A recent report from the Mayo Thrombophilia Clinic focused on subsegmental pulmonary embolism (SSPE) and reported similar results. Incidental SSPE has similar clinical outcomes to symptomatic SSPE in terms of the recurrence rate, mortality rate, major bleeding incidence, and clinically related non-major bleeding incidence [16]. It is worth noting that the study conducted by the Mayo Thrombophilia Clinic included only patients with SSPE, in which only 58.0% were provoked by active cancer [16].

Our study revealed a lower rate of recurrent VTE and similar risk of bleeding events in patients with IPE compared to those with SPE. Moreover, patients with IPE had a much better prognosis than those with SPE in both short- and long-term survival.

Since patients with IPE are at a similar or even higher risk of bleeding as well as a lower risk of recurrent VTE, it is unclear whether patients with IPE should be treated with less aggressive anticoagulation therapy, including a shorter period and/or a smaller dose of anticoagulation therapy.

Several studies have shown that cancer patients with IPE have a worse prognosis than those without PE [28]. A 1:2 case-control study conducted at MD Anderson Cancer Hospital by Odaisat et al. showed that IPE events were associated with poor outcomes in patients with cancer. The authors argued that patients with IPE should be treated with proper management plans similar to their symptomatic counterparts [28].

In addition, patients with IPE not treated with anticoagulant therapy had a worse prognosis than those treated with anticoagulant therapy. A recent pooled analysis included 926 patients with IPE from 11 cohorts and reported that the weighted pooled 6-month risks of recurrent VTE and major bleeding were 5.8% and 4.7%, respectively, with a pooled 6-month mortality of 37% [29]. The risk of recurrent VTE was comparable under LMWH (6.2%) and vitamin K antagonists (VKAs) (6.4%), both of which were much lower than that in untreated patients (12%) [29]. Additionally, the weighted pooled 6-month mortality rate was higher in untreated patients (47%) than in patients treated with LMWH (37%) and VKAs (28%).

In the absence of good quality evidence on the management of IPE, most guidelines have suggested that IPE should be treated with the same initial and long-term anticoagulation therapy as SPE. The latest National Comprehensive Cancer Network guidelines for cancer-associated venous thromboembolic disease recommend the same treatment protocol as SPE, except that outpatient management could be considered for patients with IPE [30, 31]. The American College of Chest Physicians recommends the same management, and it suggests that low-risk patients with subsegmental PE could be managed by observation, especially when PE is incidental and isolated [32]. The American Society of Clinical Oncology recommends that IPE and incidental DVT should be treated in the same manner as the symptomatic types, while treatment of incidental SSPE should be determined on a case-by-case basis [33]. The American Society of Hematology suggests that patients with IPE could be treated with 3–6 months of short-term treatment under observation [34].

Previous studies have indicated an association between being overweight and better survival outcomes in patients with cancer [35, 36]. A recent study showed that weight (per kilogram) was associated with better overall survival under adjusted multivariable analysis (HR = 0.96, 95% CI: 0.92–0.99, P = 0.032) in cancer patients with IPE [37]. However, the study was missing height data; thus, the BMI was not calculated. Our study showed that overweight was an independent protective factor in patients with cancer complicated by IPE and confirmed the previous study.

Tumor stage IV metastasis is associated with worse survival for cancer patients with PE, as shown in many studies [37]. In a separate analysis of IPE and SPE, we found that stage IV disease seemed to affect IPE more than SPE with a much higher HR. Overweight was independent protective factor associated with better prognosis in IPE patients, while independent risk factors for SPE included age, ECOG PS, and thrombolytic therapy. The aforementioned results seem to indicate that the long-term survival of IPE patients is affected by similar factors of cancer patients without PE, whereas the prognosis of SPE is more affected by the presence of PE.

Thrombolytic therapy was shown to be an independent risk factor for worse outcomes among patients with PE and those with SPE in our study. This finding may be explained by the fact that patients who received thrombolytic therapy tended to have more massive PEs and more serious situations.

Our study has some limitations. First, as this was a retrospective study, it was difficult to avoid limitations in data collection. We used electronic medical records and a database of the radiology system at Peking University Cancer Hospital to identify PEs, which helped us avoid the omission of data to the greatest extent. Also, clinical and management data were double recorded and cross-examined by two physicians to ensure the precision and completion of the data. Further, the sample size of this study was small, and certain events, such as bleeding events, were rare, which may have resulted in a type II error. Additionally, since our institution is an oncology hospital, patients from our center tended to go to other hospitals to deal with comorbidities such as VTE. In this situation, information about the switching of anticoagulant therapy, discontinuing medication, the duration of treatment, and anticoagulant efficacy was missing for some patients due to the retrospective nature of this study. Besides, although our center treated patients from different provinces, patients mainly came from North and Northeast China. This study’s results may not be generalizable to patients in other demographic areas. Finally, despite the data showing that IPE had a better prognosis than SPE, we cannot draw further conclusions that patients with IPE could receive less aggressive anticoagulant therapy than their symptomatic counterparts without detailed therapeutic data since previous literature has shown that IPE yielded a worse prognosis than cancer patients without PE [28]. Further studies are warranted to determine the appropriate treatment strategies for patients with IPE to balance thrombolytic and bleeding risks.

## Conclusions

We found that IPE comprised almost half of the PEs in patients with cancer. IPEs tended to be central, isolated, and unilateral, and they had a much better prognosis in terms of short-term and long-term survival after diagnosis than SPEs. Independent risk factors related to the long-term prognosis of patients with PE included older age, advanced disease, the presence of SPE, poor ECOG PS, and thrombolytic therapy. The location and number of arteries involved did not affect the prognosis of PE. Further research into IPE is warranted not only with regard to cancer prognosis but also in terms of indications and methods of anticoagulation.

## Data Availability

The data used in this study are available from corresponding author on reasonable request.

## Abbreviations

IPE: incidental pulmonary embolism
SPE: symptomatic pulmonary embolism
PE: pulmonary embolism
CT: computed tomography
VTE: venous thromboembolism
CTPA: CT pulmonary angiography
DVT: deep venous thrombosis
IQR: interquartile ranges
LMWH: low molecular weight heparin
ECOG PS: Eastern Cooperative Oncology Group performance status
SSPE: subsegmental pulmonary embolism
VKAs: vitamin K antagonists.
